# Next-Generation Sequencing (NGS) in non-small cell lung carcinoma: A real-world experience in the public health system of Galicia (Northwest Spain)

**DOI:** 10.1371/journal.pone.0326336

**Published:** 2025-07-01

**Authors:** Ihab Abdulkader-Nallib, José Manuel Cameselle-Teijeiro, Iván Lesende-Rodríguez, Raquel Pérez-Becerra, José Ramón Antúnez-López, Jorge García-González, Luis León-Mateos, María Sánchez-Ares

**Affiliations:** 1 Department of Pathology, Clinical University Hospital of Santiago de Compostela, Health Research Institute of Santiago de Compostela, Galician Healthcare Service (SERGAS), Santiago de Compostela, Spain; 2 School of Medicine, University of Santiago de Compostela, Santiago de Compostela, Spain; 3 University of A Coruña, A Coruña, Spain; 4 Department of Medical Oncology, Clinical University Hospital of Santiago de Compostela, Galician Healthcare Service (SERGAS), Santiago de Compostela, Spain; 5 ONCOMET, Health Research Institute of Santiago de Compostela (IDIS), Santiago de Compostela, Spain; 6 The Spanish Biomedical Research Centre in Cancer (CIBERONC), Madrid, Spain; Kore University of Enna: Universita degli Studi di Enna 'Kore', ITALY

## Abstract

The validation of several predictive biomarkers has improved the clinical outcomes of non-small cell lung carcinoma (NSCLC) patients. Single tests do not cover the mutational co-occurrences, so they do not detect other alterations, which in many cases are responsible for disease progression. We describe the development and implementation of a customized next generation sequencing (NGS) panel. We analyzed 236 formalin-fixed paraffin-embedded (FFPE) NSCLC samples from the Clinical University Hospital of Santiago de Compostela (Galicia, Northwest Spain) in 2020. Detection of *EGFR*, *KRAS*, *NRAS*, *BRAF* mutations and *ALK*, *ROS1* rearrangements were determined by real-time polymerase chain reaction (RT-PCR), immunohistochemistry (IHC) and fluorescence in situ hybridization (FISH). These results were compared with those obtained by the NGS panel to evaluate the performance of the NGS method and to identify potential novel mutations. Ten discrepancies between NGS and the orthogonal methods were found: 2 cases in the *EGFR* gene, 1 in the *KRAS* gene, 5 in the *BRAF* gene and 2 in the *ALK* gene. The most prevalent pathogenic alterations detected by NGS were: *TP53* (48.7%), *KRAS* (23.7%), *STK11* (9.7%), *EGFR* (8.5%), *PIK3CA* (5.5%), *CDKN2A* (4.7%), *BRAF* (3.4%) and *MET* exon skipping 14 (3%); rearrangements were found in *ALK* and *RET* (3.5% and 1.7%, respectively). 41.5% of NSCLC patients are harbored co-occurring mutations. Our findings confirmed the robustness, sensitivity and specificity of NGS compared to conventional approaches. NGS has a role not only in the detection of actionable alterations (including concurrent mutations), but also in stratifying patients for therapy.

## Introduction

In the current era of precision medicine, the validation of several predictive biomarkers has dramatically improved the clinical outcomes of advanced cancer patients. This concept has revolutionized the diagnosis and therapy management of diseases, with great strides made in the field of oncology [1–3]. Lung cancer remains the leading cause of cancer mortality worldwide. Non-small cell lung cancer (NSCLC) accounts for around 84% of all lung cancer cases [4,5]. The treatment landscape for NSCLC has rapidly evolved and become associated with several addictive oncogenic driver alterations. The assessment of several specific alterations, including epidermal growth factor receptor (*EGFR*), anaplastic lymphoma kinase (*ALK*), ROS proto-oncogene 1 (*ROS1*), B-Raf proto-oncogene (*BRAF*), rearranged during transfection (*RET*), hepatocyte growth factor receptor (*MET*), receptor tyrosine-protein kinase erbB-2 (*ERBB2/HER2*), Kirsten rat sarcoma viral oncogene homolog (*KRAS*), neuregulin (*NRG1*), fibroblast growth factor receptor (*FGFR*) and neuro- trophic tyrosine receptor kinase (*NTRK*) genes, are now recommended in all patients with newly diagnosed NSCLC. The guidelines of several scientific groups (College of American Pathologists [CAP], International Association for the Study of Lung Cancer [IASLC], Association for Molecular Pathology Guideline [AMP]) suggest that molecular testing should be completed at the time of a diagnosis of advanced disease or recurrence [6–8].

Improvements in the understanding of the molecular biology of NSCLC development have led to its categorization into molecular subtypes that facilitate the use of targeted therapies [7]. Co-existence of mutations may have important consequences regarding treatment. Although initially many of these driver alterations were thought to be mutually exclusive, increasing evidence shows this may not be so. Single tests do not cover the mutational co-occurrences, but such alterations could be responsible for tumor progression [9–11].

Characterizing genomic aberrations in tumors for predictive and prognostic purposes by means of genome sequencing has become an integral part of the precision medicine approach [12]. Until recently, most techniques used for this purpose included low- and medium-throughput traditional techniques such as Sanger sequencing, pyrosequencing, real time polymerase chain reaction (RT-PCR), fluorescence in situ hybridization (FISH) or immunohistochemistry (IHC). On the other hand, the study of comprehensive molecular typing of neoplasias is limited by the amount of cytological samples and/or formalin-fixed paraffin-embedded (FFPE) tissue samples, as well as by the response time required for the targeted evaluation of different genes.The increased discovery rate of clinically relevant biomarkers due to extensive characterization of cancer genomes has also necessitated the testing of multiple genes per tumor as the standard of care. Unfortunately, the aforementioned traditional technologies are unable to meet this demand. The revolutionary next-generation sequencing (NGS) technologies provide a viable alternative given their massively parallel sequencing capability, which enables the simultaneous screening of multiple genes in multiple samples [13]. Furthermore, the CAP, National Comprehensive Cancer Network (NCCN) and the European Society for Medical Oncology (ESMO) have proposed the use of expanded panels for broader molecular analysis to identify in NSCLC patients, rare driver mutations for which effective drugs are already available or under development in clinical trials [7,14–16].

Here we describe the development and implementation of CHUS-LUNG, a hybridization capture-based NGS panel capable of detecting all protein coding mutations, copy number variations (CNVs), selected promoter mutations and structural rearrangements in 48 NSCLC associated genes from FFPE samples of NSCLC. The results obtained with this NGS panel were compared with those obtained by traditional techniques, such as RT-PCR, FISH and IHC, to evaluate the performance of the NGS method and to identify novel mutations.

## Materials and methods

### Patients

We analyzed 236 FFPE tumor tissue specimens NSCLC received in the Department of Pathology at the Clinical University Hospital of Santiago de Compostela (Galicia, Northwest Spain) from March 2020 to December 2020, consecutively, from all patients who underwent molecular study according to the clinical guides of our institution [7,14–16]. The clinico-pathological features of these patients are summarized in Table 1. The mean age of the patients (165 males and 71 females), was 65.9 years, ranging from 36 to 88 years. A majority of patients (231; 97.8%) had stage IV disease, and the majority of tumors were adenocarcinomas (161; 68.2%). Sample types included 167 (70.8%) small biopsies (core and/or bronchoscopic biopsies), 59 (25%) cell blocks (endobronchial ultrasound-guided fine-needle aspiration and pleural effusion), 7 (2.9%) surgical samples and 3 (1.3%) cytological extensions.

**Table 1:** Main epidemiological and clinicopathological characteristics of the NSCLC patients.

*Age at diagnosis (years)*	65.99 (range, 36–88)
*Sex*	
Male	165 (69.9%)
Female	71 (30.1%)
*Histological subype*	
Adenocarcinoma	161 (68.2%)
Squamous cell carcinoma	58 (24.5%)
Neuroendocrine carcinoma	3 (1.3%)
Adenosquamous carcinoma	3 (1.3%)
NSCLC, NOS	10 (4.2%)
Sarcomatoid carcinoma	1 (0.5%)
*Stage*	
IV	231 (97.8%)
IIIA	4 (1.7%)
IIIB	1 (0.5%)
*Sample type*	
Small biopsy	167 (70.8%)
Surgical sample	7 (2.9%)
Cell block	59 (25%)
Cytological extension	3 (1.3%)

NSCLC, non-small cell lung cancer; NOS, not otherwise specified.

The study was carried out following the recommendations of the Declaration of Human Rights, the Conference of Helsinki [17] and the protocol of the study was approved by the Hospital Ethics Committee (code nº: 2019/629; date: 02/18/2020). Signed informed consent was obtained from all patients.

### Workflow

FFPE blocks were cut into 5 μm thick tissue sections for molecular analysis in the following order: first hematoxylin and eosin (H&E) stained slide; 3 slides prepared for immunohistochemistry and FISH analysis; 5–10 tissue sections for DNA extraction and a final H&E stained slide (S1 Fig). For all FFPE samples, an experienced pathologist, identified and marked on the second H&E the tumor rich regions of interest to avoid the risk of false negative results. All FFPE samples were scored for tumor cell percentage by standard histological assessment. The percentage of tumor content ranged from 10 to 90%. Tissue material was macrodissected using the H&E slide as a guide, and DNA was extracted from FFPE samples and cytological extensions using Qiagen’s Deparaffinization Solution (catalog number 19093; Qiagen, Hilden, Germany) and the QIAamp DNA FFPE Tissue kit (catalog number 56404; Qiagen, Hilden, Germany) according to the manufacturer’s protocol. The only modification made was to the QIAamp FFPE tissue protocol which used an overnight lysis incubation time at 56°C instead of the suggested 1 hour.

For *EGFR*, *KRAS*, *NRAS* and *BRAF* study by traditional methods, the DNA quantity and quality obtained from the samples were analysed spectrophotometrically (NanoDrop 1000, Thermo Scientific, Waltham, MA). The quality of extracted DNA was evaluated using an absorbance ratio of 260 nm to 280 nm (A260/A280). For NGS, DNA quality (DNA Integrity Number, DIN) and quantity, of the same DNA, were measured using the Genomic DNA ScreenTape system on the 4200 TapeStation system (catalog numbers 5067–5365 and 5067–5366; Agilent, Santa Clara, CA) according to the manufacturer. Samples with less than 10% of tumor cells, DIN equal to 0 and a concentration ≤ 1 ng/µl were excluded for the NGS study.

### Detection of *EGFR, KRAS, NRAS, BRAF* mutations and *ALK*, *ROS1* rearrangements

*EGFR* mutation status was determined using the RT-PCR based Cobas EGFR mutation test v2 (catalog number 07248563190; CE-IVD, Roche Diagnostics, Basel, Switzerland) according to the manufacturer’s protocol. This kit can identify 42 different *EGFR* mutations in exons 18, 19, 20, and 21, using the Roche Cobas Z480 thermocycler (Roche Diagnostic, Basel, Switzerland). *KRAS*, *NRAS* and *BRAF* mutation status was determined using the KRAS v2 mutation test (LSR) (catalog number 078927001) and BRAF/NRAS mutation test (LSR) (catalog number 07659962001) kits in the Roche Cobas z480 system (Roche Diagnostic, Basel, Switzerland) according to the manufacturer’s instructions. These RT- PCR tests examined the most common mutations in codons 12, 13, 59, 61, 117 and 146 in the *KRAS* and *NRAS* genes; and in codons 466, 469, 600 and 601 of the *BRAF* gene. Data analysis was performed by uploading files to the online LSR Data Analysis tool https://lifescience.roche.com/en_nl/brands/oncology-research-kits.html.

A third method was used to further analyze some of the samples having a discordant result between the RT-PCR and NGS assays. The Idylla™ EGFR Mutation Test (catalog number A0060/6), an automatic cartridge-based RT-PCR assay, was performed using the Biocartis Idylla™ System (Biocartis, Mechelen, Belgium), according to the manufacturer’s indications, in the cases with discordant results in *EGFR*. *KRAS* and *BRAF* genes were reanalyzed using the Therascreen KRAS Pyro Kit (catalog number 970460) and Therascreen BRAF Pyro Kit (catalog number 971470) (Qiagen, Hilden, Germany) according to manufacturer’s recommendation, respectively. Pyrosequencing was performed on the PyroMark Q24 platform (Qiagen, Hilden, Germany) using the PyroMark Gold Q24 reagents. Pyrograms were generated with PyroMark Q24 software (v.2.0.6.) and the data were analyzed manually or with a plug-in tool provided by Qiagen (Qiagen, Hilden, Germany).

*ALK* and *ROS1* rearrangements were studied by IHC employing the VENTANA ALK (D5F3) CDx Assay (catalog number 790−4796; Roche Diagnostics, Basel, Switzerland) and IHC ROS1 Clone D4D6 (catalog number 3287S; Cell Signaling Technology, Danvers, MA, USA), respectively. Positive IHC ALK assays were confirmed by fluorescence in situ hybridization employing Vysis ALK Break Apart FISH Probe kit (catalog number 06N38-023; Abbot, Abbot Park, IL).

### Design of the custom cancer panel

Custom RNA probes in this study were designed for targeted sequencing for all exons, 3´UTR (unstranlated region) and 5´UTR regions and at least, 25 bp 5′ and 3′ flanking intronic sequences of 48 NSCLC-related genes for detection of single-nucleotide variants (SNVs), insertions and deletions (indels), and CNVs: *AKT1* (AKT serine/threonine kinase 1, NM_05163), *AKT3* (AKT serine/threonine kinase 3, NM_005465), *ALK* (NM_004304), *APC* (APC regulator of WNT signaling pathway, NM_000038), *BRAF* (NM_004333), *BRCA1* (BRCA1 DNA repair associated, NM_007294), *BRCA2* (BRCA2 DNA repair associated, NM_000059), *CDKN2A* (cyclin dependent kinase inhibitor 2A, NM_000077), *CTNNB1* (catenin beta 1, NM_001904), *DDR2* (discoidin domain receptor tyrosine kinase 2, NM_001014796), *EGFR* (NM_005228), *ERBB2* (NM_004448), *ERBB3* (erb-b2 receptor tyrosine kinase 3, NM_001982), *ERBB4* (erb-b2 receptor tyrosine kinase 4, NM_005235), *FGFR1* (fibroblast growth factor receptor 1, NM_023110), *FGFR2* (fibroblast growth factor receptor 2, NM_000141), *FGFR3* (fibroblast growth factor receptor 3, NM_000142), *HRAS* (HRas proto-oncogene, GTPase, NM_00534), *IDH1* (isocitrate dehydrogenase (NADP(+)) 1, NM_005896), *IDH2* (isocitrate dehydrogenase (NADP(+)) 2, NM_002168), *JAK1* (Janus kinase 1, NM_002227), *JAK2* (Janus kinase 2, NM_004972), *KEAP1* (kelch like ECH associated protein 1, NM_203500), *KIT* (KIT proto-oncogene, receptor tyrosine kinase, NM_000222), *KRAS* (NM_033360), *MAP2K1* (mitogen-activated protein kinase kinase 1, NM_002755), *MAP2K2* (mitogen-activated protein kinase kinase 2, NM_030662), *MET* (NM_000245.), *MYC* (MYC proto-oncogene, bHLH transcription factor, NM_002467), *NF1* (neurofibromin 1, NM_00026), *NRAS* (NRAS proto-oncogene, GTPase, NM_002524), *NRG1* (NM_013962), *NTRK1* (neurotrophic receptor tyrosine kinase 1, NM_002529), *NTRK2* (neurotrophic receptor tyrosine kinase 2, NM_006180), *NTRK3* (neurotrophic receptor tyrosine kinase 3, NM_001012338), *PDGFRA* (platelet derived growth factor receptor alpha, NM_006206), *PIK3CA* (phosphatidylinositol-4,5-bisphosphate 3-kinase catalytic subunit alpha, NM_006218), *POLD1* (DNA polymerase delta 1, catalytic subunit, NM_002691), *POLE* (DNA polymerase epsilon, catalytic subunit, NM_006321), *PTEN* (phosphatase and tensin homolog, NM_000314), *RB1* (RB transcriptional corepressor 1, NM_000321), *RET* (NM_020975), *ROS1* (NM_002944), *SLC34A2* (solute carrier family 34 member 2, NM_006424.3), *SOX2* (SRY-box transcription factor 2, NM_003106), *STK11* (serine/threonine kinase 11, NM_000455), *TERT* (telomerase reverse transcriptase, NM_198253), *TP53* (tumor protein p53, NM_000546). In addition, the panel was also designed to identify rearrangements by detecting the intronic breakpoints previously described in 11 genes (*ALK, RET, ROS1, NTRK1, NTRK2, NTRK3, NGR1, FGFR1, FGFR2, FGFR3* and *BRAF* genes). The oligonucleotides are 120 bp in length and were manufactured by Agilent-developed sureprint technology, the software to create the 93856 probes was SureDesign (Agilent, Santa Clara, CA) which uses different algorithms to create the probes based on the percentage of Guanine-Cytosine (GC) and the repetitive regions of the genome. The user only needs to select the genomic region and the software creates the probes to ensure the best possible capture of area of interest. The probes are 5’biotinylated oligonucleotide. After isolating probes annealed to complementary fragments with streptavidin-coated beads, the library fragments were sequenced by adding an adapter compatible with this sequencing system (Illumina, Inc, San Diego CA). This means that it was not the probes that were sequenced, but the regions that were complimentary to these probes. This strategy makes it possible to detect fusions of these genes with any other partner throughout the genome. The panel includes the main actionable biomarkers relevant for the therapy, prognosis, and diagnosis of NSCLC, as well as other genes associated with therapies being tested in clinical trials or susceptible to being developed in the future in several solid tumor types.

The genes were selected based on clinical guidelines such as National Comprehensive NCCN Guidelines®, ESMO and the National Consensus of the Spanish Society of Pathology and the Spanish Society of Medical Oncology [18], as well as recommendations of international organizations such as the Food & Drug Administration (FDA) and the European Medicines Agency (EMA).

### Library construction and sequencing

All reactions were performed in the range of 50–200 ng of the total genomic DNA (gDNA) that was fragmented in the Covaris M220 focused-ultrasonicator (Covaris Inc., Woburn, MA) to generate DNA fragments between 150–200 bp. Libraries were prepared using the SureSelect XT HS Target Enrichment system using the Magnis NGS Prep system (catalog number G9771D; Agilent, Santa Clara, CA) following the manufacturer’s protocol. Libraries were validated and quantified using the High Sensitivity D1000 ScreenTape system on the 4200 TapeStation system (catalog numbers 5067–5584 and 5067–5585; Agilent, Santa Clara, CA) according to the manufacturer’s protocol. Pooled libraries were subsequently sequenced using the NextSeq 550 Mid Output Kit v2.5 (150 cycles) on the Illumina NextSeq 550 system as 2 × 76-bp paired-end reads (catalog number 20024904; Illumina, Inc., San Diego, CA), running 24 samples for each sequencing run to obtain an average coverage of about 400x (>95% of the gene’s target nucleotides are covered at 200x, after removing the duplication reads). This platform collects all the information in demultiplexed and paired FASTQ files for subsequent bioinformatic analysis.

### Data analysis and classification of variants

The obtained FASTQ files were aligned to the NCBI human reference genome (hg19-Genome Reference Consortium GRCh37). The alignment and variant calling were assessed through SureCall software v.4.2 (Agilent, Santa Clara, CA), with interpretation and priorization by Alissa Interpret Analysis Software v.5.3.4 (Agilent, Santa Clara, CA) using a filtering tree. CNVs were identified using open source software called CNVkit [19]. Variants were visually examined using the Integrative Genome Viewer (IGV) from the Broad Institute (http://www.broadinstitute.org/igv). In order to assess the pathogenicity of the detected sequence variants several databases were used: Catalogue of Somatic Mutations in Cancer (COSMIC, http://cancer.sanger.ac.uk/cosmic), Single Nucleotide Polymorphism Database (dbSNPs, http://www.ncbi.nlm.nih.gov/snp), NCBI’s Clinical Variants (ClinVar, http://www.ncbi.nlm.nih.gov/clinvar), My Cancer Genome (MCG, http://www.mycancergenome.org) and Oncology Knowledge Base (OncoKB, https://www.oncokb.org/).

### Analytical validation: Sensitivity, specificity and reproducibility analysis

The validation analysis was performed using 18 well-characterized samples to evaluate the presence or absence of somatic variants. The sensitivity and specificity were determined by comparing NGS testing with conventional methods for routinely tested alterations. All mutations were detected, resulting in an analytical sensitivity of 100%. No other mutations were detected in the total of 18 samples analyzed, thus resulting in an analytical specificity of greater than 99%. To calculate reproducibility, the closeness between the results of successive analyses of the same panel carried out under different measurement conditions was analyzed. The conditions that changed in the reproducibility tests were: a) the origin of the sample, b) the operator who performed the test, and c) the reagents used and the measurement equipment with which the test was carried out. The same 3 samples were analyzed in two different assays with different operators, kits with different batches and different sequencing equipment. The 3 variants were detected in both assays, which gave a reproducibility of 100%. The minimum detection limit was 1%. To evaluate the performance of the NGS assay for a minimum detection limit of 1%, 3 samples with known variants were selected and diluted with a negative sample for the same variant until reaching an allelic frequency of 1%. They were analyzed in triplicate in the same assay, and in triplicate in a second assay to evaluate the reproducibility parameter. The 3 selected samples had the following mutations: *EGFR* exon 19 deletion, *ALK* translocation and p.Q61H point mutation in the *KRAS* gene. The set of mutations was detected in the 3 samples and in the two series, resulting in values greater than 95% for repeatability with variants at an allelic frequency of 5% reaching the minimum detection limit.

## Results

### Mutations detected by orthogonal methods and NGS

Table 2 summarizes all *EGFR*, *KRAS* and *BRAF* mutations detected by both NGS and RT-PCR. We detected mutated *EGFR* in 8.5% of NSCLC patients by targeted NGS, and by RT-PCR. 23.7% of patients had a *KRAS* gene mutation according to targeted NGS. All tumors containing an *EGFR* mutation and 91.1% of *KRAS* mutated cases were adenocarcinomas. 50% of patients carrying an *EGFR* mutation were women, whereas 73.2% of the patients with a *KRAS* mutation were men and 26.7% women. *BRAF* mutation was detected in 3.4% of patients with NSCLC according to targeted NGS and 1% by RT-PCR. Almost all samples with *BRAF* mutation (87.5%), seven out of eight, were adenocarcinomas and 75% were men. Rearrangements were found in *ALK* in eight patients (3.5%) by NGS and six patients (2.6%) by IHC/FISH, 57.2% of them women (S2 Fig). *NRAS* mutation was found in one patient by both methods. No *ROS1* rearrangement was found.

**Table 2:** *EGFR*, *KRAS* and *BRAF* mutations detectable by NGS and RT-PCR in NSCLC.

		Mutation	Single Test	Mutation	NGS
*EGFR*	Exon 18	G719X	1	p.G719A	1
		Deletion	0	p.E709_T710delinsD	1
	Exon 19	Deletion	9	p.E746_T751delinsA	1
				p.E746_A750del	4
				p.S752_I759del	1
				p.L747_E749del	2
				p.L747_A750del	1
	Exon 20	T790M	2	p.T790M	2
		Insertion	2	p.H773_V774insH	1
	Exon 21	L858R	6	p.L858R	6
*KRAS*	Codon 12	G12C	22	p.G12C	25
		G12X	22	p.G12V	13
				p.G12D	5
				p.G12S	2
				p.G12A	2
				p.G12L	1
	Codon 13	G13X	4	p.G13C	2
				p.G13D	1
	Codon 60	G60X	1	p.G60V	1
	Codon 61	Q61X	3	p.Q61H	3
	Codon 146	A146X	1	p.A146V	1
*BRAF*	Exon 11		0	p.G464V	1
				p.G469V	2
				p.G469R	1
	Exon 15	V600E/E2/D	2	p.V600E	2
				p.G596R	1
				p.K601E	1

NGS, next-generation sequencing, RT-PCR, real-time polymerase chain reaction, NSCLC, non-small cell lung cancer; NOS, not otherwise specified.

### Concordance between NGS and orthogonal methods

Table 3 shows a very high concordance of the results between NGS and RT-PCR. There were only 10 discrepancies between the NGS and the other methods. We have not identified any correlation between discordant results and clinicopathological characteristics (S1 Table). Two discordant *EGFR* mutation results were detected; one mutation (insertion exon 20) was detected by RT-PCR but not by NGS; in contrast indel p.E709_T710delinsD in exon 18 was found in NGS but not in the Cobas assay. The first sample was reanalyzed using the Idylla™ EGFR Mutation Test (Biocartis, Mechelen, Belgium) with no mutation detected. Thus, we concluded NGS to be the correct assay. In the second case, indel is not targeted in the design of the Cobas assay and was not considered discordant.

**Table 3:** Comparison of *EGFR*, *KRAS*, *NRAS*, and *BRAF m*utation and *ALK* and *ROS1* detection by NGS and conventional methods (single test) in NSCLC.

		Concordant NGS/ST		Discordant NGS/ST				
Gene	Nº of cases	-/-	+/+	-/+	+/-	Concordance (%)	Sensitivity	Specificity
*EGFR*	236	217	17	1	1	99.2	0.94	0.99
*KRAS*	67	13	53	1	0	98.5	0.98	–
*NRAS*	188	187	1	0	0	100	1	–
*BRAF*	201	195	1	1	4	97.5	0.50	0.97
*ALK*	233	226	6	0	2	99.1	0.83	0.99
*ROS1*	232	232	0	0	0	100	1	–

NGS, next-generation sequencing; NSCLC, non-small cell lung cancer; ST, single test.

One discrepancy was found in the 67 NSCLC patients tested for *KRAS*; a G13X mutation was detected by RT-PCR but not by NGS. This sample was reanalyzed using the Therascreen KRAS Pyro Kit (Qiagen, Hilden, Germany) with a negative result, thus showing a false positive detected by RT-PCR.

Five discordant BRAF mutations were detected in NSCLC patients, four mutations (1 case p.G464V, 2 cases p.G469V, 1 case p.V600E were detected by NGS but not by RT-PCR and 1 variant (V600E/E2/D) detected by RT-PCR but not by NGS. These samples were reanalyzed by pyrosequencing yielding results concordant with NGS. Mutation p.G464V is not targeted in the BRAF/NRAS mutation test design (LSR) (Roche Diagnostic, Basel, Switzerland) and was not considered discordant (S3 Fig).

Out of 233 patients tested, 6 (2,5%) were positive for ALK rearrangements in both NGS and ST and 2 were discordant. One case was analyzed by FISH and the result was positive; in the other, the study could not be carried out due to the absence of material.

**Fig 1:**
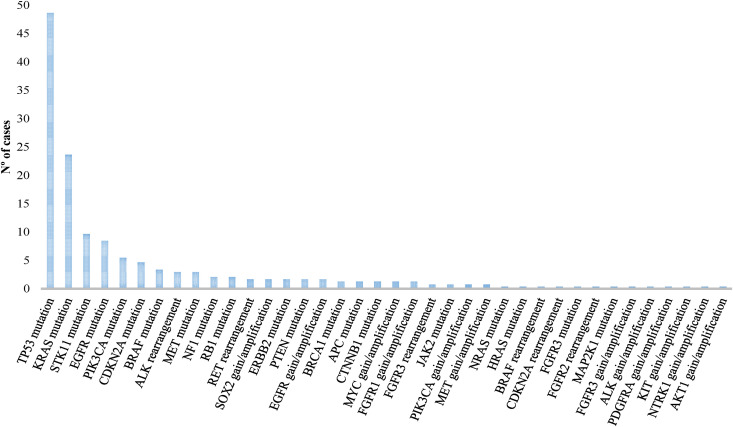
Pathogenic alterations detected by NGS in NSCLC. NGS, next-generation sequencing; NSCLC, non-small cell lung cancer.

The pathogenic alterations detected by NGS are shown in Fig 1. The most prevalently mutated gene was *TP53* (48.7%) followed by *KRAS* (23.7%), *STK11* (9.7%), *EGFR* (8.5%), *PIK3CA* (5.5%), *CDKN2A* (4.7%), *BRAF* (3,4%) and *MET* exon skipping 14 (3%). Rearrangements were found in *ALK* and *RET* (3.5% and 1.7%, respectively).

**Fig 2:**
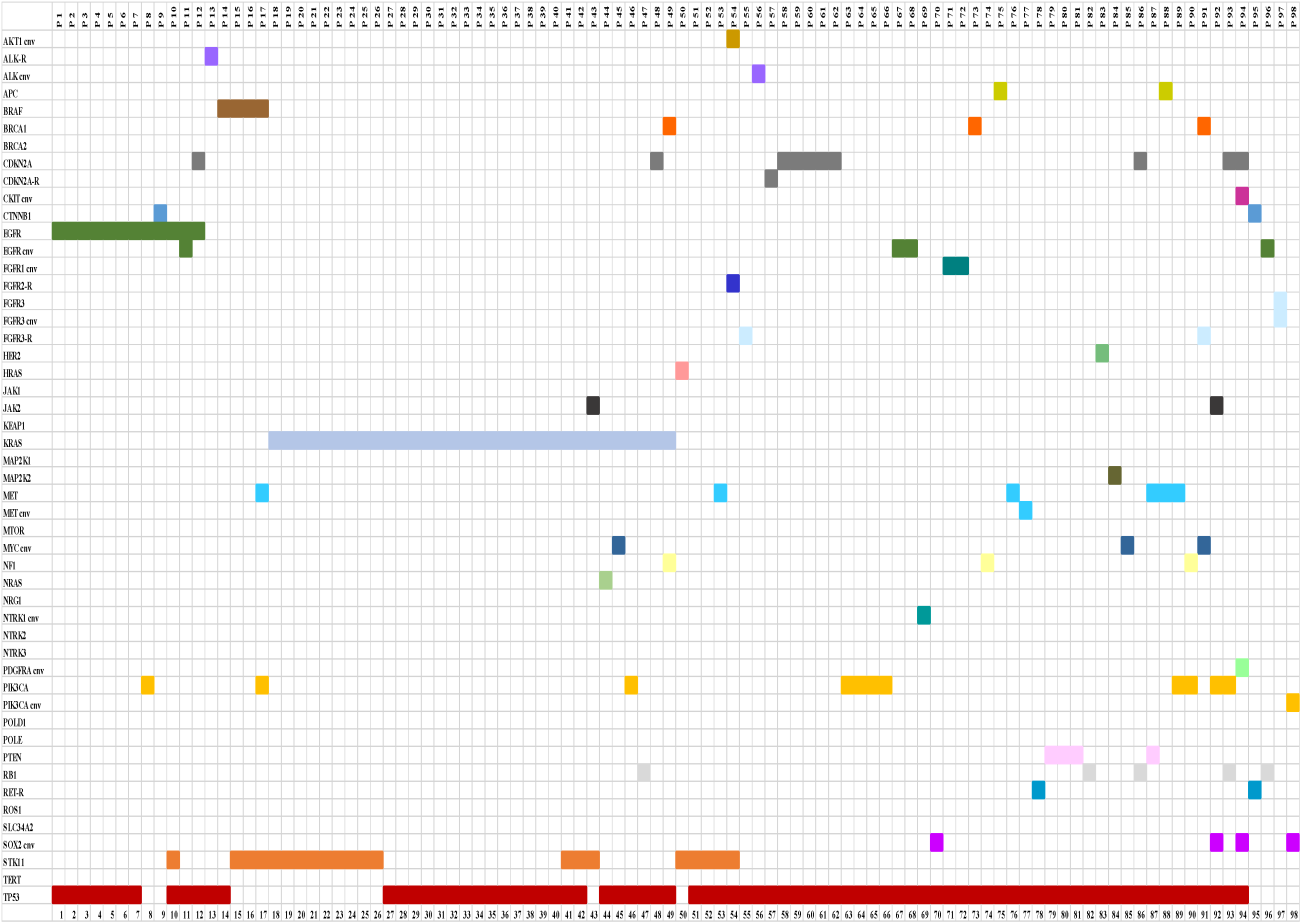
Associations among the most prevalently mutated genes in NSCLC patients. P, patient; R, rearrangement; CNV; copy number variation; NSCLC, non-small cell lung cancer.

Ninety-eight (41.5%) of the 236 NSCLC patients harbored co-occurring mutations. In 73 (74.5%) patients there were two molecular alterations and 25 (25.5%) patients had three or more. *TP53* was present in 78 of 98 co-occurring mutations. *KRAS* and *TP53* was the most frequent combination (n = 14) followed by *KRAS* and *STK11* (n = 12). Seven *EGFR* mutations appeared in concurrency with *TP53* mutations. Two patients carried a deletion in exon 19 of the *EGFR* gene accompanied by mutations in *PIK3CA* and *CTNNB1* genes, respectively. Two *EGFR* mutated patients harbored a double concurrent mutation, one with *TP53* and *STK11* mutations and the other with a *TP53* mutation and *EGFR* gain/amplification. One *EGFR* mutated patient had concurrent mutations in *TP53*, *CDKN2A* and *CTNNB1* genes. Among the 56 *KRAS* mutated samples, fourteen were in concurrency with *TP53* mutations, twelve with *STK11* mutations, seven *KRAS* with two concurrent mutations (*CDKN2A*, NRAS, *PIK3CA*, *RB1*, *STK11*, *TP53* and/or *MYC* amplification), and one *KRAS* with three concurrent mutations (*BRCA1*, *NF1* and *TP53*). *BRAF* mutation was found in concurrency with *STK11* (n = 2), *TP53* (n = 1) and one case with concurrent mutations in *STK11*, *PIK3CA* and *MET* exon 14 skipping. *NRAS* was concomitant in one sample with *KRAS* and *TP53*. One patient with *ALK* rearrangement also had a concurrent *TP53* mutation (Fig 2).

**Fig 3:**
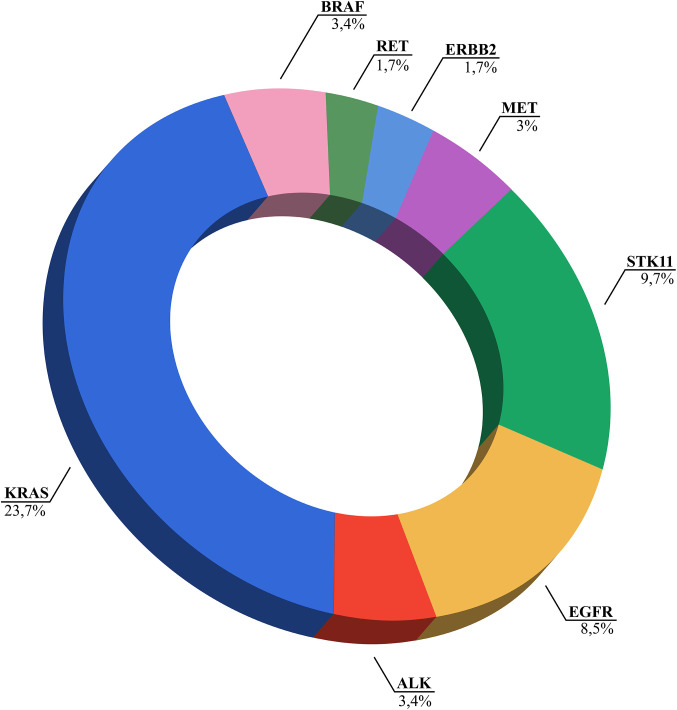
Percentage of NSCLC patients with clinically relevant actionable alterations detected by NGS. NSCLC, non-small cell lung cancer; NGS, next-generation sequencing.

NGS identified clinically relevant genomic alterations in 55% of NSCLC patients (Fig 3). The most prevalent was detected in *KRAS* (23.7%) followed by *STK11* (9.7%) and *EGFR* (8.5%). *BRAF* mutation and *MET* exon 14 skipping were detected in 3.4% and 3% respectively. Duplication and insertions exon 20 (1.7%) were found in *ERBB2*. Eight patients showed fusions between *ALK* and *EML4* (Echinoderm microtubule-associated protein-like 4), all these rearrangements involving exon 20 of *ALK*. In three patients the rearrangement included exon 13, (*EML4(13)::ALK(20)*, variant 1), in two patients exon 20 (*EML4(20)::ALK(20)*, variant 2), in one patient exon 2 (*EML4(2)::ALK(20)*, variant 5) and in another exon 6 (*EML4(6)::ALK(20)*, variant 3). Four patients presented rearrangements in *RET*, two of them involving the kinesin family member 5B (*KIF5B)* (*KIF5B(15)::RET(12)*) and the other two involving the coiled-coil domain containing 6 *(CCDC6)* (*CCDC6(1)::RET(12)*) and the ankyrin repeat and sterile alpha motif domain containing 1B *(ANKS1B)* (*ANKS1B(8)::RET(12)*), respectively.

## Discussion

The integration of a high-throughput technology into clinical routine practice of a public health system represents a major challenge [20–22]. In this study, we show that NGS technology is able to efficiently amplify and sequence multiple genes with small amounts of DNA from FFPE samples, as well as analyze different genomic alteration events (SNVs, indels, CNVs and/or fusions) (S2 Table). We screened for *EGFR*, *KRAS*, *BRAF* mutations and *ALK* and *ROS1* rearrangements in NSCLC by targeted NGS and conventional methods. Our findings confirm the excellent sensitivity and specificity of NGS compared with single-gene targeted assays. The use of these panels requires a shorter run time than sequential studies, as shown in S3 Table. In addition, these panels are cost-effective, maximize coverage, and minimize off-target effects [23–26]. Overall, concordance of our results was very high, and we detected only ten discrepant results between NGS and the orthogonal methods: two cases in the *EGFR* gene, one in the *KRAS* gene, five in the *BRAF* gene and two in the *ALK* gene.

Regarding *EGFR* mutations, we found a patient with squamous cell carcinoma who tested positive with the Cobas® assay (insertion exon 20) and not mutated by NGS. The absence of the insertion was confirmed by Idylla. The possibility of Cobas® EGFR being able to generate false positive results for exon 20 insertion mutations has been previously described. In their article, Kanaoka et al reported four Cobas® EGFR results positive for exon 20 insertion in four patients with squamous cell carcinoma that later tested negative using NGS and fragment analysis. As the authors mentioned, more studies are necessary to determine the mechanism responsible for these false positives [27]. Our results highlight the need to validate this type of alterations by a second method to select the correct treatment for the patient. In another case, an insertion in exon 18 of *EGFR* was detected by NGS but not by Cobas. This mutation is not included in the Cobas test, which highlights one of the limitations of this PCR method, which only identifies the most common loci. With advances in NGS technology, the regions of interest can be sequenced to improve the chances of identifying rare variants which are susceptible to treatment with EGFR-TKI. Rare *EGFR* mutations could also be equally important targets in the treatment of advanced-stage lung cancer; therefore, detailed information regarding *EGFR* mutation type is needed to apply appropriate therapies. These results indicate the use of targeted sequencing with improved sensitivity to identify mutations of low frequency. In our experience, the additional mutations detected by targeted sequencing were helpful to understand the clinical course of some of the patients in each group.

Concerning *KRAS* testing, we detected one discordant case. One sample was positive by RT-PCR assay but negative by NGS. This sample was re-tested using a pyrosequencing assay, which then provided results concordant with the NGS assay. The most usual technique to identify *KRAS* mutations is PCR. Thus far, their detection in isolation has not been recommended, but should be included in NGS panels [28]. At our institution, *KRAS* was not routinely tested in all patients by RT-PCR; it was only carried out later in those patients who were positive in NGS using the same DNA. After years without effective therapies, several inhibitors have been developed that have shown activity in phase II trials against the *KRAS* p.G12C mutation, such as sotorasib and adagrasib [29,30]. These drugs have been approved by the FDA, although their benefit in monotherapy or in combination, as well as the profile of the patients to which they should be administered, are being studied in phase III trials [22].

For *BRAF* mutations, there were five discordant cases between NGS and Cobas. In the NGS study, we found four patients with *BRAF* mutation non p.V600E that were negative by RT-PCR. Current approved targeted inhibitors for *BRAF* driven lung cancers include dabrafenib and vemurafenib, which are only effective toward p.V600E mutation. Nevertheless, in patients with lung cancer, approximately 70% of *BRAF* mutations are non-V600 mutations, leaving these patients without a targeted therapy. Mechanistic studies supported by structural modeling strongly suggest that although the p.G469V substitution activates BRAF, it also renders it sensitive to direct targeting by the EGFR TKIs [31]. Another study by Noeparast *et al*., investigated a patient cohort of NSCLC and demonstrated that non-V600 *BRAF* mutations, resulting in either high or impaired kinase activity, confer sensitivity to combined dabrafenib and trametinib treatment [32]. Therefore, *BRAF* testing with sequencing assays is mandatory for the correct diagnosis and treatment of patients with advanced NSCLC given that they could benefit from these treatments.

Concerning *ALK* rearrangements, in our cohort, we observed two fusions by NGS not identified by IHC. One case was reanalyzed by FISH and resulted positive; in the other case, the study could not be carried out due to the absence of material. The patients’ medical history was reviewed and in both cases the patients are being treated with ALK inhibitors and have shown response. Some of the reasons associated with false negative results are: a) the use of pre-cut slides that can cause differences in the intensity of IHC staining, at times even causing unequivocally false negative results; b) suboptimal simple fixation; c) suboptimal analytical phase, and/or d) the presence of fewer than 50 tumor cells [33]. Our data are consistent with those of *Velizheva et al*., who demonstrate the greater robustness and reliability of targeted NGS for fusion detection, compared to isolated tests such as FISH [34]. Overall, NGS has proven to be a valid alternative to conventional molecular testing in terms of diagnostic accuracy [35].

Mutations in the *EGFR* gene are identified in approximately 10–16% of NSCLCs, with a higher frequency among patients with adenocarcinoma and non-smoking patients. We found *EGFR* gene mutations in 8.5% of patients, in line with frequencies reported in Spain and western countries, when taking into consideration that in our series 69.9% were men and only 68.2% adenocarcinomas. *EGFR* mutation subtypes p.L858R and deletions in exon 19 were the most frequent in our cohort in accordance with what is described in other articles [10,36,37]. Mutations in *KRAS* are identified in 25% of patients with NSCLC. They are found in all histological subtypes of adenocarcinoma, although more common in the invasive mucinous subtype [38]. In our series, 23.7% of patients have *KRAS* mutations, and mutations p.G12C, p.G12V, and p.G12D were the most frequent, fitting well with previous reports [39]. For *BRAF* mutations, our NGS study revealed a percentage of 2.5%, similar to that reported in the literature [36,40]. *ALK* rearrangements are present in 3.5% of patients in our study, which is within the 2–5% previously reported [41,42]. Our percentage of 1% of *NRAS* is the same as that found in other series of the Spanish population [36]. No *ROS1* rearrangements were detected in this study, which can be explained by the epidemiological and clinico-pathological characteristics of our series together with the low prevalence of *ROS1* rearrangement reported in NSCLC [43,44].

Our study has shown concurrent mutations in 41.5% of patients, which exemplifies the complexity of the molecular biology of NSCLC. This is a rather high proportion in comparison to another series, which previously reported at a frequency of 2–12.3% [10,45,46] and also higher when compared to another study in a Spanish population, which was 35% [36]. In some cases, the co-existence occurs among non-activating mutations, but frequently it involves well known activating mutations. The rate of co-existence can vary among the different techniques, depending on their sensitivity. Won et al found a 4.4% *EGFR* and *ALK* co-existence frequency in *ALK* positive patients screened by Sanger sequencing. This was further raised to a 15% frequency when high sensitivity NGS methodology was applied [47]. Importantly, our study uncovered an underappreciated utility of NGS testing. *EGFR* and *TP53* co-mutation was associated with poorer progression free survival (PFS) outcomes when treated with first-line EGFR TKI than when compared with a single EGFR mutation. This was especially seen in *EGFR* p.L858R mutation patients, with no significant difference in *EGFR* exon 19 deletion patients [24,48]. *STK11* mutations are present in variable percentages in different tumor types. The frequency of *STK11* mutations in lung cancer varies from 5% to 30%. In a real-world study by Shire NJ et al. [49], a prevalence of 13.6% for mutated *STK11* was found, with co-occurring *KRAS* mutations in 6.5% of patients. The prevalence of *STK11* in our study was 9.7%, which is within the range previously reported. About half (5.1%) of the patients with *STK11* mutations had a co-mutation in *KRAS*, which is also consistent with the frequency of co-mutations previously reported in the literature [49]. Co-existence of other oncogenic alterations such as *ERBB2* and *MET* amplifications and *PIK3CA*, *PTEN* and *RB1* mutations have also been associated with poorer responses to therapy [11]. Additionally, NGS has the benefit of detecting the specific fusion variant and partner, which in *ALK* rearranged NSCLC, may have prognostic and therapeutic implications [9].

A limitation of the present study has been the lack of data available to establish a correlation between the molecular alterations and the treatment of the patients. The NGS panel is limited to 48 genes. Nor is it possible to establish any correlation between stage and type of mutations. Another additional limitation of this work is that it has not been designed to compare the response time between orthogonal methods and NGS.

In summary, 55% of NSCLC patients harbored a potential clinically actionable alteration, consistent with previous studies [36,50] and according to current guidelines [7,16,22], confirming the applicability of these technologies in precision medicine. Multiple biomarker testing has become a major challenge for molecular diagnostic laboratories given the increasing number of approved targeted. NGS therefore has a role, not only in detecting actionable alterations, but also in stratifying patients for therapy.

## Supporting information

S1 Table Clinico-pathological characteristics of discordant results. NGS, next-generation sequencing; M, male; F, female.
(DOCX)

S2 Table Main characteristics of the customized CHUS-LUNG panel compared to commercial panels. (DOCX)

S3 Table Comparison of the main advantages and disadvantages between NGS and conventional sequencing methods. NGS, next-generation sequencing.
(DOCX)

S1 Fig Diagram of the workflow of samples from patients with non-small cell lung carcinoma (NSCLC). First, the tumor areas were identified and marked, quantifying the percentage of tumor cells. Then, 3 slides were prepared for immunohistochemistry (IHC) and fluorescence in situ hybridization (FISH). Next, DNA extraction was carried out, including quality and quantity controls. Finally, the orthogonal and next generation sequencing (NGS) studies were carried out. In cases with discordant results, an additional alternative methodology was used.
(TIF)

S2 Fig Immunohistochemical and fluorescence in situ hybridization (FISH) images in non-small cell lung carcinoma (NSCLC). (A) Lung adenocarcinomas showing immunohistochemical positivity (brown staining) (A) and negativity (B) for A*LK*. FISH image showing *ALK* rearrangements (arrows)(C). *ALK* FISH negative specimen (D). Immunohistochemical negativity for ROS1 in a case of the present series (E), and positive control for ROS1 (F). Lung adenocarcinomas with and without *ALK* and *ROS1* rearrangements have been used as positive and negative controls, respectively.
(TIF)

S3 Fig Pyrograms of *KRAS* (codon 13) and *BRAF* (codons 469 and 600) of discordant results between NGS and RT-PCR assays. The left column shows the results of the pyrosequencing studies that are consistent with those obtained by NGS. The right colum shows the negative and positive controls. Wild-type codon 13 of *KRAS* gene (A) and positive control of mutated codon 13 of *KRAS* (arrow) (B). Pyrogram showing a mutation in codon 469 of *BRAF* gene (arrow) (C), and wild-type control of codon 469 of *BRAF* (D). Pyrogram showing a mutation in codon 600 of *BRAF* gene (arrow) (E), and wild-type control of codon 600 of *BRAF* gene (F). Wild-type codon 600 of *BRAF* gene (G), and positive control of mutated codon 600 of *BRAF* gene (arrow) (H).
(TIF)
