# Supplementary material for: Next-Generation Sequencing (NGS) in non-small cell lung carcinoma: A real-world experience in the public health system of Galicia (Northwest Spain)
Source: PLoS One. 2025 Jul 1;20(7):e0326336. doi: 10.1371/journal.pone.0326336 (PMC12212532; doi:10.1371/journal.pone.0326336)
Supplement: S1 Table — NGS, next-generation sequencing; M, male; F, female. (DOCX) [file pone.0326336.s001.docx]

| Table S1. Clinico-pathological characteristics of discordant results. | | | | | | |
| --- | --- | --- | --- | --- | --- | --- |
|  | | | | | | |
| **Case** | **Analitical**  **method** | | **Clinicopathological**  **characteristics** | | | |
|  | *Ortogonal*  *method* | *NGS* | *Histological subtype* | *Stage* | *Age*  *at diagnosis* | *Sex* |
| 1 | Insertion exon 20 *EGFR* | No mutated | Squamous cell carcinoma | IV | 63 | F |
| 2 | G13X *KRAS* | No mutated | Adenocarcinoma | IV | 56 | F |
| 3 | No mutated | p.G469V *BRAF* | Adenocarcinoma | IV | 65 | M |
| 4 | No mutated | p.G469V *BRAF* | Adenocarcinoma | IV | 57 | F |
| 5 | No mutated | p.V600E *BRAF* | Adenocarcinoma | IV | 81 | M |
| 6 | V600E/E2/D *BRAF* | No mutated | Adenocarcinoma | IV | 47 | F |
| 7 | IHC negative *ALK* | *EML4::ALK* | Adenocarcinoma | IV | 59 | M |
| 8 | IHC negative *ALK* | *EML4::ALK* | Adenocarcinoma | IV | 53 | F |
| NGS, next-generation sequencing; M, male; F, female. | | | | | | |
