# Supplementary material for: Next-Generation Sequencing (NGS) in non-small cell lung carcinoma: A real-world experience in the public health system of Galicia (Northwest Spain)
Source: PLoS One. 2025 Jul 1;20(7):e0326336. doi: 10.1371/journal.pone.0326336 (PMC12212532; doi:10.1371/journal.pone.0326336)
Supplement: S2 Table — (DOCX) [file pone.0326336.s002.docx]

| Table S2. Main characteristics of the customized CHUS-LUNG panel compared to commercial panels. | | | |
| --- | --- | --- | --- |
|  | | | |
|  | **CHUS-LUNG** | **FoundationOne CDx (Foundation Medicine)** | **Oncomine Dx Target Test (ThermoFisher Scientific)** |
| Number of genes | 48 | 324 | 23 |
| Type of sequencing | DNA seq | DNA seq | DNA seq/RNA seq |
| Underlying technology | Hybrid-capture | Hybrid-capture | Amplicon |
| Input material | 50 ng DNA | 50 ng DNA | 10 ng DNA/10 ng RNA |
| Sequencing platform | Illumina | Illumina | Ion PGM Dx sequencing system |
