# Supplementary material for: Next-Generation Sequencing (NGS) in non-small cell lung carcinoma: A real-world experience in the public health system of Galicia (Northwest Spain)
Source: PLoS One. 2025 Jul 1;20(7):e0326336. doi: 10.1371/journal.pone.0326336 (PMC12212532; doi:10.1371/journal.pone.0326336)
Supplement: S3 Table — NGS, next-generation sequencing. (DOCX) [file pone.0326336.s003.docx]

| Table S3. Comparison of the main advantages and disadvantages between NGS and conventional sequencing methods. | | | |
| --- | --- | --- | --- |
| **Method** | **Advantages** | **Disadvantages** | **TAT** |
| *IHC* | Widely available | Indirect demostration of the fusion gene | 2-3 days |
|  | Low cost | Occasional false negative results |  |
|  | High sensitivity and specificity | Subjective interpretation of results |  |
|  | Automated test | Variability depend on fixation procedure, antibody clones and detection methods |  |
| *FISH* | High specificity | Lower sensitivity | 2-3 days |
|  | Low input of material | Technical challenges (signal instability and scoring) |  |
|  | This method allows the detection of unknown fusions | Precise annotation of the fusion variant is not possible. |  |
|  | Established in many labs | Difficult to interpret (requieres diagnostic expertise) |  |
| *RT-PCR* | Well-established method | Only detects the mutations included in the test. | 3-4 days |
|  | Rapid diagnostic method |  |  |
|  | High sensitivity and specificity |  |  |
|  | Easier interpretation of the results. |  |  |
| *Pyrosequencing* | High accuracy | Short length of sequence | 3-4 days |
|  | Provides sequence information | Costly |  |
|  | Determines relative mutational load | Difficult to interpret some alterations |  |
| *NGS* | Simultaneous screening of multiple genes in multiple samples | High complexity of workflow | 5-6 days |
|  | Better picture of tumor heterogeneity | Evaluation and interpretation of higher-complexity data |  |
|  | Higher discovery rate of new markers | The use of NGS is not very widespread in pathology laboratories |  |
| TAT, timearound time; IHC, immunohistochemistry; FISH, fluorescence in situ hybridization; RT-PCR, real-time polymerase chain reaction; NGS, next-generation sequencing. | | | |
