# Supplementary figures and images for: Next-Generation Sequencing (NGS) in non-small cell lung carcinoma: A real-world experience in the public health system of Galicia (Northwest Spain)
Source: PLoS One. 2025 Jul 1;20(7):e0326336. doi: 10.1371/journal.pone.0326336 (PMC12212532; doi:10.1371/journal.pone.0326336)

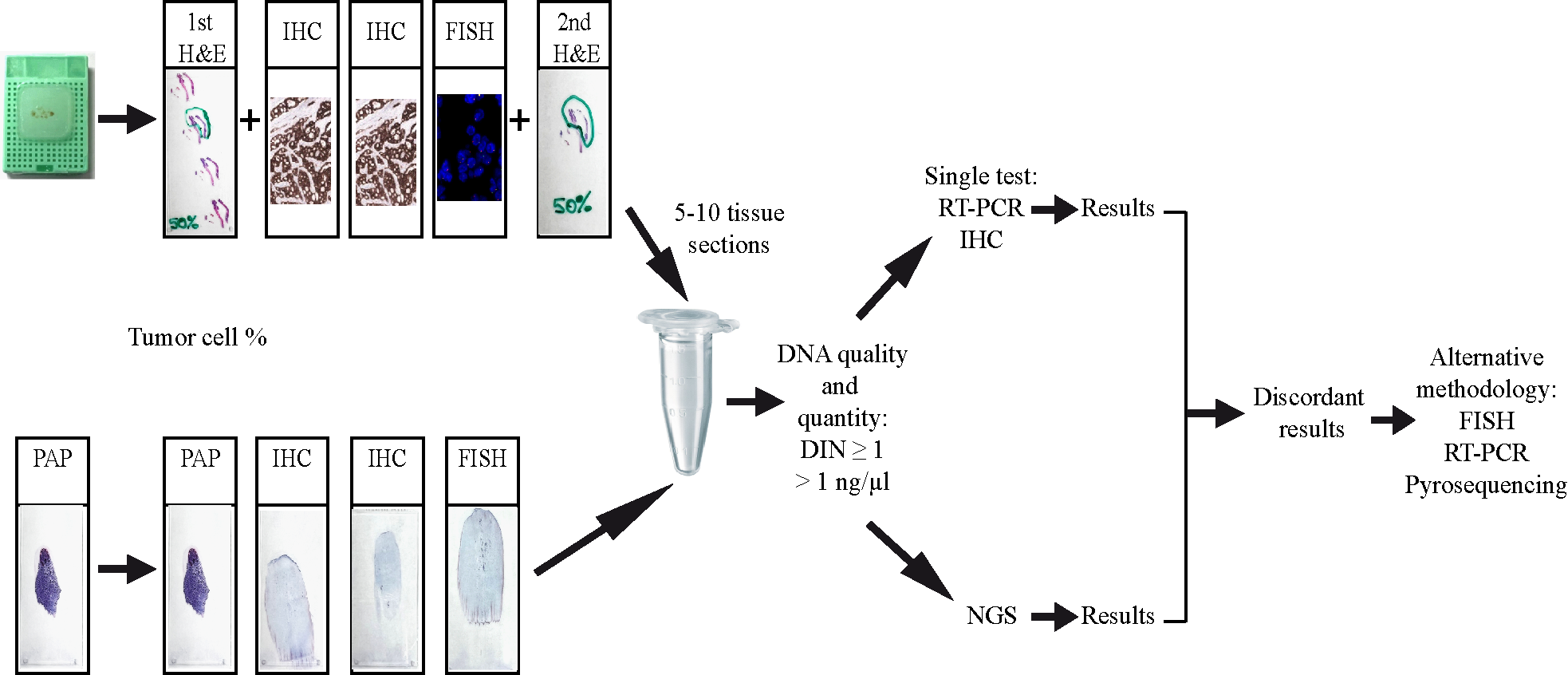

Supplement: S1 Fig — First, the tumor areas were identified and marked, quantifying the percentage of tumor cells. Then, 3 slides were prepared for immunohistochemistry (IHC) and fluorescence in situ hybridization (FISH). Next, DNA extraction was carried out, including quality and quantity controls. Finally, the orthogonal and next generation sequencing (NGS) studies were carried out. In cases with discordant results, an additional alternative methodology was used. (TIF) [file pone.0326336.s004.tif]

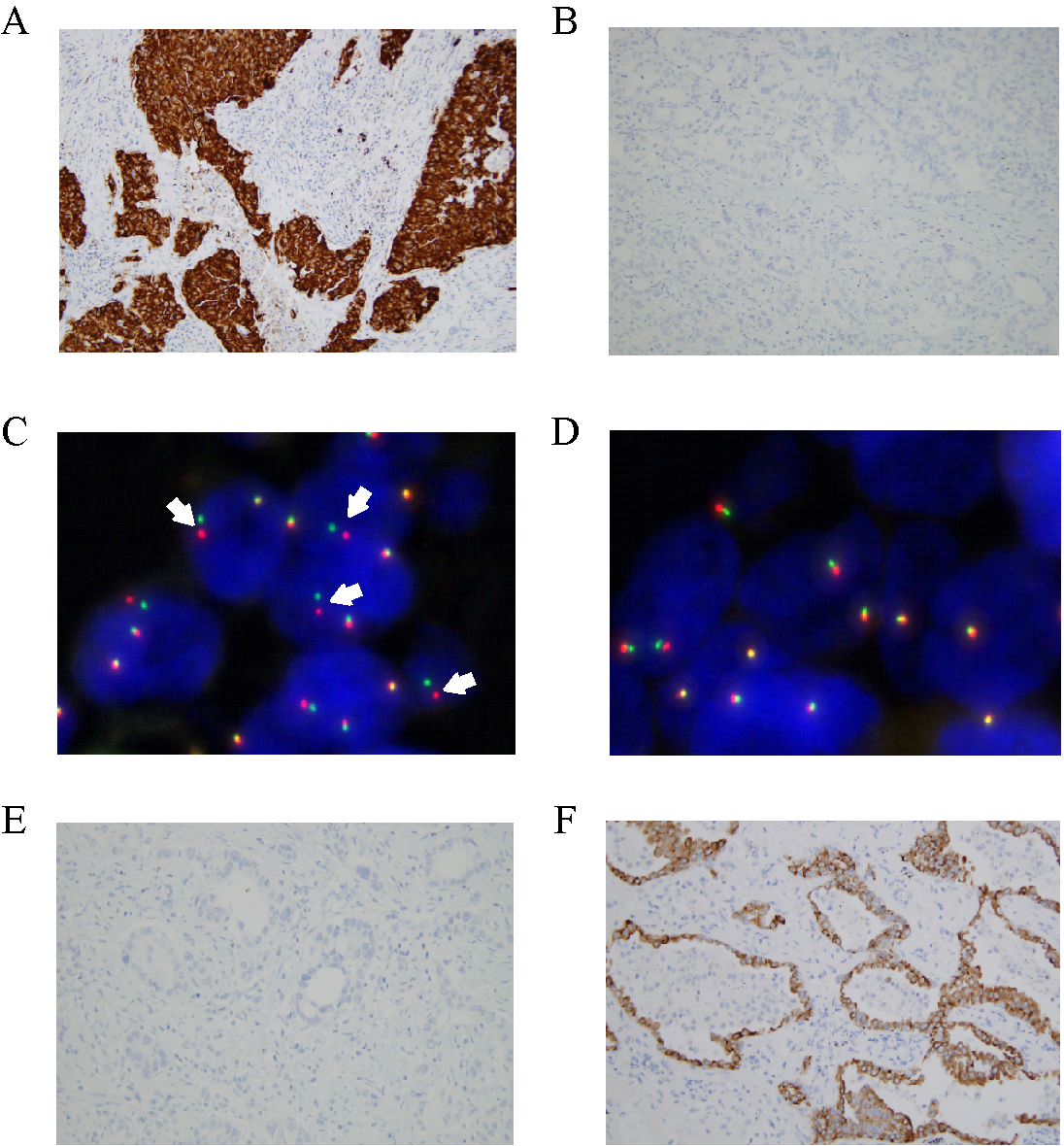

Supplement: S2 Fig — (A) Lung adenocarcinomas showing immunohistochemical positivity (brown staining) (A) and negativity (B) for ALK. FISH image showing ALK rearrangements (arrows)(C). ALK FISH negative specimen (D). Immunohistochemical negativity for ROS1 in a case of the present series (E), and positive control for ROS1 (F). Lung adenocarcinomas with and without ALK and ROS1 rearrangements have been used as positive and negative controls, respectively. (TIF) [file pone.0326336.s005.tif]

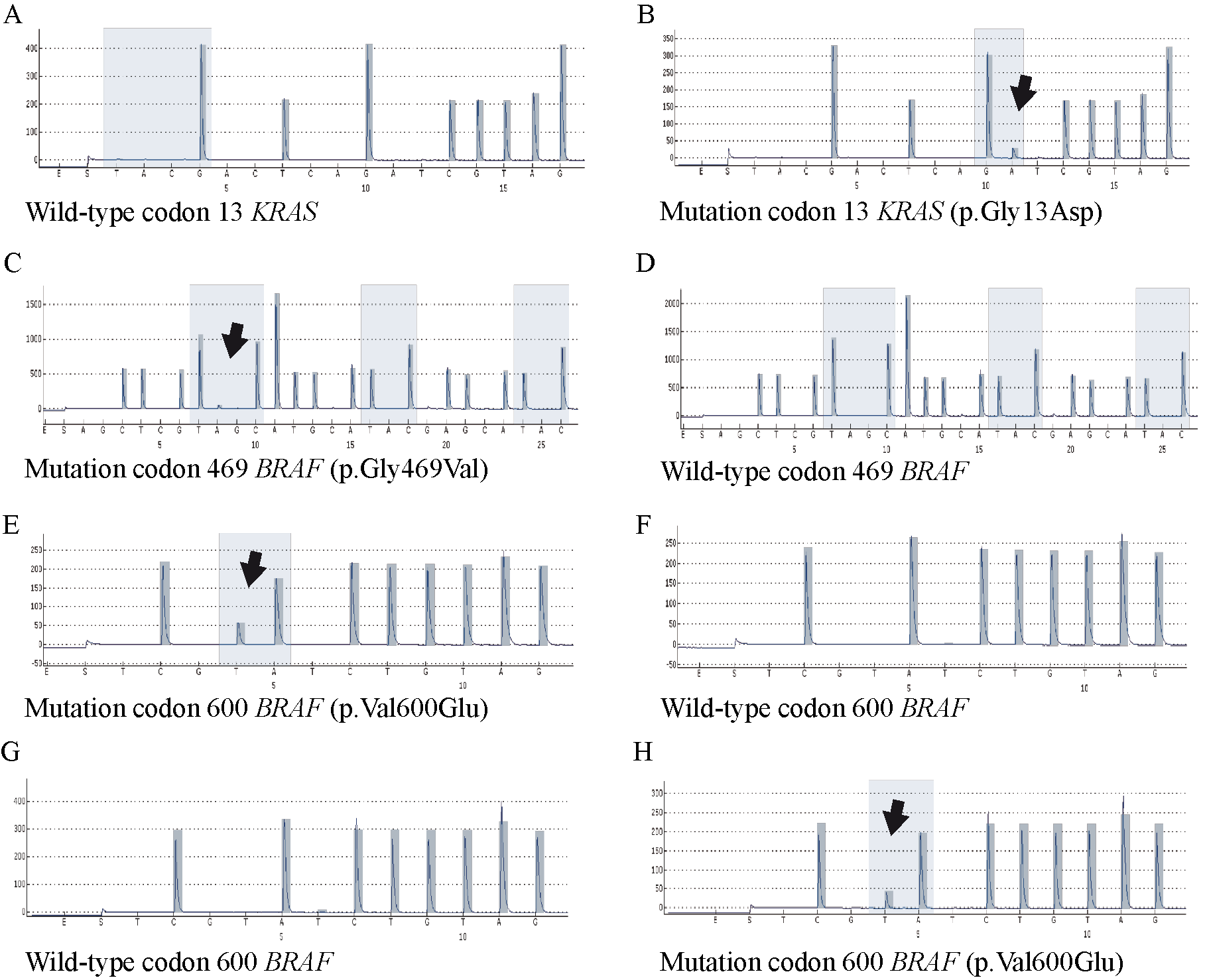

Supplement: S3 Fig — The left column shows the results of the pyrosequencing studies that are consistent with those obtained by NGS. The right colum shows the negative and positive controls. Wild-type codon 13 of KRAS gene (A) and positive control of mutated codon 13 of KRAS (arrow) (B). Pyrogram showing a mutation in codon 469 of BRAF gene (arrow) (C), and wild-type control of codon 469 of BRAF (D). Pyrogram showing a mutation in codon 600 of BRAF gene (arrow) (E), and wild-type control of codon 600 of BRAF gene (F). Wild-type codon 600 of BRAF gene (G), and positive control of mutated codon 600 of BRAF gene (arrow) (H). (TIF) [file pone.0326336.s006.tif]
